# miR-514a regulates the tumour suppressor NF1 and modulates BRAFi sensitivity in melanoma

**DOI:** 10.18632/oncotarget.3924

**Published:** 2015-04-23

**Authors:** Mitchell S. Stark, Vanessa F. Bonazzi, Glen M. Boyle, Jane M. Palmer, Judith Symmons, Catherine M. Lanagan, Christopher W. Schmidt, Adrian C. Herington, Robert Ballotti, Pamela M. Pollock, Nicholas K. Hayward

**Affiliations:** ^1^ QIMR Berghofer Medical Research Institute, Herston, Brisbane, QLD, Australia; ^2^ School of Biomedical Sciences, Institute of Health and Biomedical Innovation, Queensland University of Technology, Brisbane, Australia; ^3^ Inserm U1065, Centre Méditerranéen de Médecine Moléculaire, Equipe 1, Biologie et pathologies des mélanocytes, Nice, France

**Keywords:** miRNA, microRNA, miR-514α, BRAFi, NF1

## Abstract

To identify ‘melanoma-specific’ microRNAs (miRNAs) we used an unbiased microRNA profiling approach to comprehensively study cutaneous melanoma in relation to other solid malignancies, which revealed 233 differentially expressed (≥2 fold, p < 0.05) miRNAs. Among the top 20 most significantly different miRNAs was hsa-miR-514a-3p. miR-514a is a member of a cluster of miRNAs (miR-506-514) involved in initiating melanocyte transformation and promotion of melanoma growth. We found miR-514a was expressed in 38/55 (69%) melanoma cell lines but in only 1/34 (3%) other solid cancers. To identify miR-514a regulated targets we conducted a miR-514a-mRNA ‘pull-down’ experiment, which revealed hundreds of genes, including: *CTNNB1*, *CDK2*, *MC1R*, and *NF1*, previously associated with melanoma. *NF1* was selected for functional validation because of its recent implication in acquired resistance to *BRAFV600E*-targeted therapy. Luciferase-reporter assays confirmed *NF1* as a direct target of miR-514a and over-expression of miR-514a in melanoma cell lines inhibited NF1 expression, which correlated with increased survival of *BRAFV600E* cells treated with PLX4032. These data provide another mechanism for the dysregulation of the MAPK pathway which may contribute to the profound resistance associated with current RAF-targeted therapies.

## INTRODUCTION

Melanoma is a multifaceted disease that exhibits all the usual ‘hallmarks’ of cancer [1] shared across a wide range of malignancies. However, despite these similarities in the tumourigenic process, most cancers exhibit a set of characteristics unique to the tumour or the tissue/cell of origin.

microRNAs (miRNAs) are ~22 nucleotide long sequences that are central regulators of gene expression and can act both in a positive and a negative way to control protein levels in the cell. If a miRNA binds with perfect complementarity to its target mRNA, then AGO2 is directed to cleave the mRNA, which leads to mRNA degradation. However, in most cases the binding of the mRNA to the target gene is imperfect, which leads to silencing of the gene by preventing or reducing translation. As miRNAs primarily bind imperfectly to mRNAs, they have the potential to bind to and regulate the functions of thousands of genes. Due to these intrinsic characteristics, a single miRNA or ‘cluster’ of miRNAs has the ability to control gene expression across a multitude of different signaling pathways. In melanoma, numerous miRNAs have been implicated in tumour development [2] and some have been shown to being relatively ‘tissue-specific’. This notion of miRNA tissue specificity was first explored by Gaur *et al* [3] using the NCI-60 cell line cancer panel. Even with only eight melanoma cell lines included, it was evident miRNA expression was able to discriminate melanoma from other cancer types. Since this seminal paper, there have been a limited number of studies that have harnessed the power of miRNA for melanoma diagnostics [4-6], although some have utilized next-generation sequencing for the discovery of ‘tissue-specific’ miRNAs [7, 8].

In the current study we sought to expand our current understanding of ‘melanoma-specific’ miRNA expression by performing a comprehensive microarray analysis of melanoma compared to a range of other solid tumour types, and to follow up key microRNAs which may be critical to aspects of melanoma biology.

Here, in the largest study to date, we identified a significant number of miRNAs (*n* = 233) with known and novel relationships to melanoma. One particular miRNA, miR-514a-3p (miR-514a) is a member of a cluster of miRNAs on chrXq27.3 that has been implicated in the malignant transformation of melanocytes, along with the promotion of tumour formation [9]. Using a ‘pull-down’ procedure [10, 11] we revealed hundreds of miR-514a-regulated genes including some melanoma associated genes: *CTNNB1*, *CDK2*, *MC1R*, and *NF1*. We subsequently confirmed that miR-514a has the ability to bind to *NF1* and inhibit its translation. Furthermore, we show that miR-514a expression is involved in modulating cell proliferation rates along with BRAFi (PLX4032) sensitivity in melanoma.

## RESULTS

### ‘Melanoma-specific’ miRNA discovery profiling in melanoma

Comprehensive analysis of a large panel of melanoma cell lines identified distinct ‘tissue-specific’ miRNA expression compared to other solid malignancies. We used miRNA microarrays (miRBaseV18) to study the miRNA profile of cutaneous melanoma (*n* = 55), uveal melanoma (*n* = 7), and RNA derived from melanoma patient serum/plasma (*n* = 3) in relation to ‘other’ solid cancers (*n* = 34). Normal pigment cells (melanocytes and melanoblasts) and pre-malignant cells (nevocytes) were also included as controls (‘Discovery cohort’). In this ‘Discovery’ set, a total of 233/1898 differentially expressed (≥2 fold, *p* < 0.05; See Materials and Methods) miRNAs were identified when ‘melanomas’ were compared to ‘other’ cancers (Supp Figure 1 and Supp Table 1). Supp Table 2 summarizes the top up- and down-regulated miRNAs (by at least 10-fold), which have either known or novel relationships with melanoma.

### ‘Lineage-specific’ miRNA validation

Notably, the top two miRNAs up-regulated in melanoma were hsa-miR-211-5p (miR-211) and hsa-miR-514a-3p (miR-514a) with profound average fold-changes of 276 and 204 respectively compared to other solid malignancies (Supp Tables 1 and 2). It is well known that miR-211 is deemed to be a lineage specific miRNA [12] however, we provide the first reported evidence that miR-514a also fits into this category. Supp Table 1 shows relatively high expression of miR-514a in melanocytes (MELA) and melanoblasts (QF1160MB), no detectable expression in a congenital nevus (MM653), detectable expression in 69% and 43% of cutaneous and uveal melanoma cell lines respectively, and expression in only 1/34 ‘other’ solid cancers. To test the robustness of the array data, quantitative real-time PCR validation was performed with these miRNAs along with a selection of other miRNAs from the array (Supp. Figure 2 and Supp Table 3). Supp. Figure 2 highlights that the array and real-time PCR data are well correlated.

### MITF regulation of ‘lineage-specific’ miRNAs

**Figure 1:**
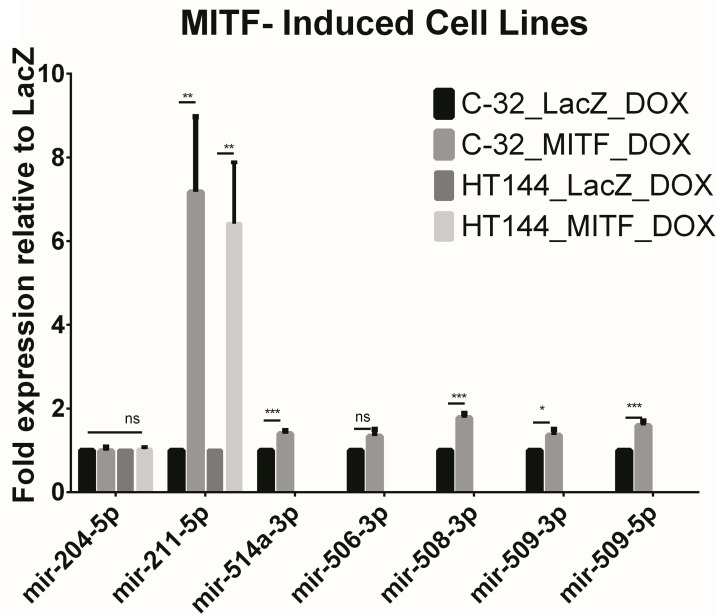
Figure shows the miRNA expression of the known lineage-specific miRNA, miR-211-5p along some other highly expressed miRNAs with known associations with melanoma In MITF-inducible melanoma cell lines (C-32 and HT144), miR-211-5p expression was significantly upregulated as compared to LacZ induced cell lines. Members of the miR-506-514 cluster show only mild upregulation in MITF-induced C-32 when endogenous levels were expressed which indicates that the cluster is likely to be independently regulated. No basal or induced expression was observed in HT144 (MITF or LacZ). Error bars represent SD from four technical replicates. ns = non-significant. *p < 0.05, **p < 0.01, ***p < 0.005.

MITF is a melanocyte lineage specific transcription factor which has previously been shown to regulate miR-211 levels via its host gene *TRPM1* [13]. As miR-514a is part of a melanocyte-specific cluster of miRNAs [9] and is highly expressed along with miR-211 levels, we sought to investigate whether this tissue-specificity could be considered MITF-dependent. Using an inducible MITF system [14] we measured the expression levels of miR-514a along with other highly expressed lineage-specific miRNAs; miR-204-5p, miR-211-5p, mir-506-3p, miR-508-3p, miR-509-3p, and miR-509-5p. miR-211 was confirmed as being under transcriptional control of MITF following MITF induction in two melanoma cell lines (C-32 and HT144). The 6-7-fold increase in miR-211 in these cell lines following MITF induction was statistically significant (*p* = 0.01; Figure 1). Some of the miR-506-514 cluster members had modest (1.4-1.8 fold) but significantly increased expression after MITF induction in the C-32 cell line (miR-514a; *p* = 0.001, miR-508-3p; *p* = 0.0003, miR-509-3p (*p* = 0.05), and miR-509-5p (*p =* 0.002)), however they were not significantly increased in HT144. We thus conclude that the effect observed in C-32 is likely to be indirect, rather than through direct transcriptional regulation of the miR-506-514 cluster by MITF. Additionally, miR-204 which differs by one nucleotide to miR-211 is regulated independently to miR-211 as there was no difference in expression following induction with MITF (Figure 1).

### Target gene identification via biotin-labeled miRNA duplex ‘pull-down’ of mRNA transcripts

Due to the high levels of miR-514a in melanoma compared to other cancers, we sought to identify its specific target genes by performing a whole-genome mRNA expression array following a biotin-labeled miRNA duplex pull-down of bound mRNAs (See Materials and Methods). This comprehensive approach revealed hundreds of genes that were ‘pulled-down’ as compared to a negative scrambled control miRNA duplex (Neg-Scr)(Supp Table 4). Supp. Figure 3 shows the lists of genes associated with the key words: ‘MELANOMA’, ‘BRAF’, and ‘MITF’; along with the genes common to all. The common genes pulled-down by miR-514a included *ATF4*, *BIRC3*, *CDK2*, *CREB1*, *CTNNB1*, *ERBB3*, *EZH2*, *FOS*, *IL18*, *KDM5B*, *MC1R*, *NF1, SPRY4*, *STAT1*, *TSC1*, and *TSC2*.

### Dual-luciferase reporter assays confirm binding of miR-514a to NF1

**Figure 2:**
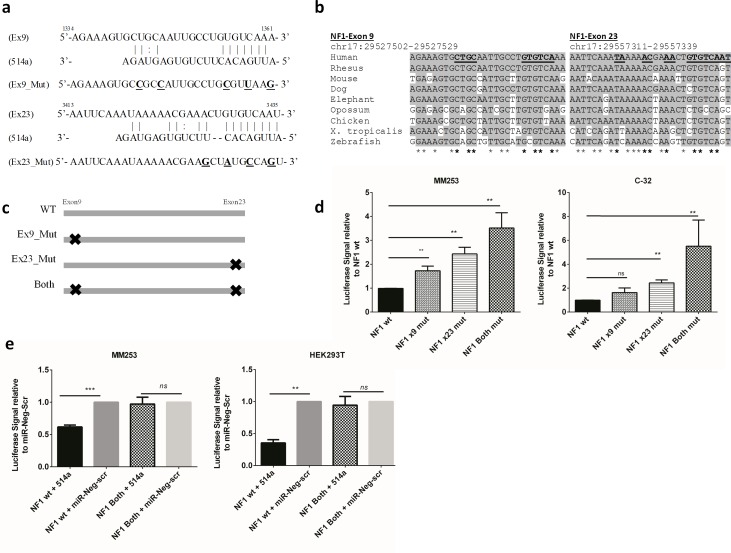
**a.** Schematic diagram of the two predicted miR-514a-3p binding sites in Exon 9 (Ex9) and Exon 23 (Ex23) of *NF1.* Shown underneath Ex9 and Ex23 are the mutations made in the NF1 cDNA fragment for luciferase assays (bolded and underlined). **b.** Multiple species alignment of the miR-514a binding. Underlines = miR-514a binding sites. Shading and stars = nucleotide conservation present in the alignment. **c.** Diagram of mutations generated in NF1 cDNA fragment. Black crosses = mutation of binding site. **d.** Dual-Luciferase Reporter (DLR) assays. MM253 and C-32 were transfected with constructs shown in panel **C**, as well as pGL4.75 [hRluc/CMV] to normalize the data. (**e**) MM253 and HEK293T were co-transfected with miR-514a mimic and miR-Neg-scr together with NF1 wildtype and mutant constructs (Both). Error bars show standard error of the mean (SEM) from a triplicate experiment; ns = non-significant. **p < 0.01, ***p < 0.005.

In order to validate the robustness of the ‘pull-down’ assay we selected *NF1* for follow up and sought to confirm direct binding of miR-514a to the transcript. Using the miRanda prediction algorithm, the top two highly conserved (Figure 2a-2b) putative binding sites of miR-514a were located in the coding region of *NF1* (NM_000267), in exons 9 and 23 respectively (see Materials and Methods and Figure 2). Coding region binding is not commonly reported in the literature but an increasing number of studies attest to its relevance e.g. [15-17]. As dogma suggests, there were binding sites in the 3′UTR but these were below the default binding threshold (binding threshold = 140) of the prediction algorithm, along with 79 (binding threshold = 100) other potential binding sites across the length of the NF1 transcript (NM000267) (Supp Table 5). It was not feasible to assess all of the binding sites so we chose to focus on the best hits. To assess the function of these binding sites, a cDNA sequence spanning this region was cloned upstream of a luciferase reporter construct. Mutation of either the first, or the second, or both putative miR-514a-binding sites (Figure 2c) led to an increase in reporter activity in MM253 and C-32 (Figure 2d) cells compared to the reporter containing the cloned wild-type NF1 sequence which relied on endogenous miR-514a expression. Figure 2d shows that both binding sites are required and have an additive effect on recovery of luciferase signal (mean 3.5-fold increase in signal compared to wild-type). To further confirm that miR-514a was involved, a miR-514a mimic along with a negative control (miR-Neg-scr) was co-transfected with NF1-mutant constructs (both sites) (Figure 2c). Figure 2e highlights that overexpressed miR-514a successfully bound to the NF1 wild-type construct, which resulted in a reduction in luciferase signal. Furthermore, mutation of both of these sites prevented binding of miR-514a in both MM253 and a non-melanoma cell line, HEK293T (which has no endogenous expression of miR-514a) (Figure 2e). These data provide strong evidence for miR-514a direct binding to NF1 mRNA.

### Inhibition of miR-514a increases protein expression of NF1 along with increased cell proliferation

**Figure 3:**
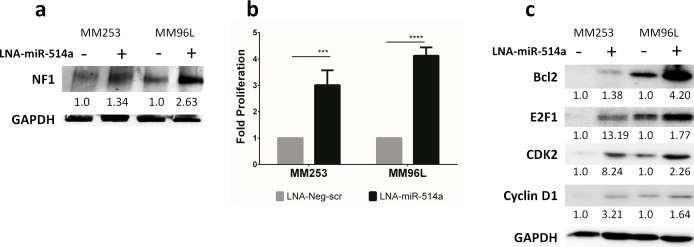
**a.** NF1 expression is increased following transfection in with a miR-514a inhibitor (LNA-miR-514a) as compared to a negative scrambled control (LNA-Neg-scr). NF1 relative expression is normalized to the loading control (GAPDH). Numbers indicated relative fold change. NF1 (~300kDa) and GAPDH (37kDa) were run and quantified on the same gradient gel **b**. MM253 and MM96L were transfected with LNA-miR-514a and LNA-Neg-scr with proliferation measured at day 6 post transfection. **c**. Cell cycle (E2F1, CDK2, and Cyclin D1) and apoptosis-related (BCL-2) proteins are increased following transfection with LNA-miR-514a as compared LNA-Neg-scr. All proteins are normalized to the loading control (GAPDH) and compared to their respective negative control. Numbers indicated relative fold change. E2F1 (70kDa), CDK2 (33 kDa), Cyclin D1 (36kDa) and GAPDH (37kDa). Error bars show standard error of the mean (SEM) from 2 biological triplicate experiments; *P* values as indicated, ***p<0.005, **** *p*<0.001.

We selected melanoma cell lines (MM253 and MM96L) that had detectable NF1 protein to assess if NF1 expression could be altered following manipulation of miR-514a. Inhibition of miR-514a with a Locked-Nucleic Acid (LNA) resulted in an increase of NF1 protein expression (1.3-2.6 fold higher) (Figure 3a) compared to a negative srambled control (LNA-Neg-scr). We next sought to investigate the effect of inhibition of miR-514a on cell proliferation given the prior reports on growth rate and increased NF1 expression. Contrary to the expected result, we found that inhibition of miR-514a led to a significant increase in cell proliferation (Figure 3b) in MM253 (p = 0.002; mean 3 fold) and MM96L (*p* = 3.17e-008; mean 4 fold) as measured by SRB assay.

### Inhibition of miR-514a increases protein expression of CDK2 and other cell cycle progression markers

To investigate why we observed a marked increase cell proliferation even in the presence of increased NF1 protein, we selected an additional gene for target confirmation (CDK2; Supp Table 4). CDK2 also showed increased expression upon inhibition of miR-514a (2.3-8.2 fold higher) (Figure 3c). As CDK2 is a well known cell cycle progression marker, this prompted an investigation into additional cell cycle and apoptosis markers. Indeed, measurement of the cell cycle proteins E2F1 (1.8-13.2 fold higher) and cyclin-D1 (1.6-3.2 fold higher), and the anti-apoptosis marker, BCL2 (1.4-4.2 fold higher), revealed that these too were increased upon inhibition of miR-514a (Figure 3c).

### Overexpression of miR-514a leads to down-regulation of NF1 protein and a decrease in cell proliferation and colony formation

**Figure 4:**
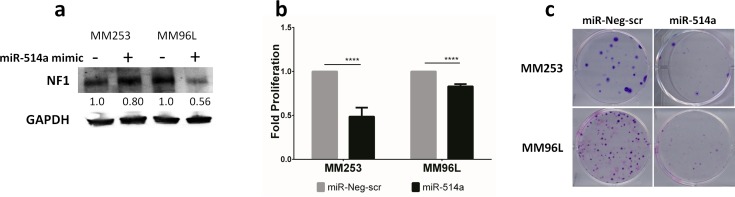
**a.** NF1 expression is reduced following transient transfection of miR-514a-3p (miR-514a) mimic (5 nM) as compared to miR-Neg-scr control. Numbers indicated relative fold change. NF1 (~300 kDa) and GAPDH (37 kDa) were run and quantified on the same gel. **b.** MM253 and MM96L were transiently transfected with miR-514a mimic (5 nM) and miR-Neg-scr with proliferation measured at day 6 post transfection. Error bars show standard error of the mean (SEM) of 3 biological triplicates; *P* values as indicated, ****P < 0.001. **c.** MM253 and MM96L were transiently transfected with miR-514a mimic (5 nM) and a negative control (miR-Neg-scr) with colonies stained with crystal violet 14 days post transfection. This assay was repeated twice in duplicate and representative results are shown.

Transient transfection of 5 nM of a miR-514a mimic, or a miR-Neg-scr (for comparison) into MM253 and MM96L, showed a reduction of NF1 protein levels (0.80- 0.56 fold) after 72 hours (Figure 4a), which was observed up to 6 days post-transfection (data not shown). Interestingly, despite a reduction in protein levels, NF1 mRNA was increased in MM96L (data not shown), whereas in MM253, the mRNA expression was reduced (data not shown), mirroring the protein expression (Figure 4a). These data indicate that the binding of miR-514a to NF1 mRNA is dynamic, and, in different cell contexts, can reduce as well as increase expression. These data also highlight that the only reliable measurement for an effect of miRNA binding is via protein expression which is in keeping with miRNAs being post-transcriptional modifiers. Nevertheless, with the combination of the luciferase assays (Figures 2d-2e) and the western blot analysis affecting NF1 expression upon miR-514a depletion (Figure 3a) and over-expression (Figure 4a), we provide strong evidence for miR-514a-mediated direct regulation of NF1 protein levels.

As inhibition of miR-514a promoted cell proliferation, we next sought to investigate the effect of overexpression of miR-514a. Indeed, cell proliferation was significantly decreased in both MM253 (*p* = 0.0005; 2.1-fold less) and MM96L (*p* = *7*.40e-005; 1.3-fold less) (Figure 4b). This was subsequently confirmed in an *in vitro* clonigenicity assay, with both cell lines having markedly reduced colony formation upon overexpression of miR-514a (Figure 4c).

### miR-514a expression levels modulate BRAFi sensitivity in BRAFV600E melanoma cell lines

**Figure 5:**
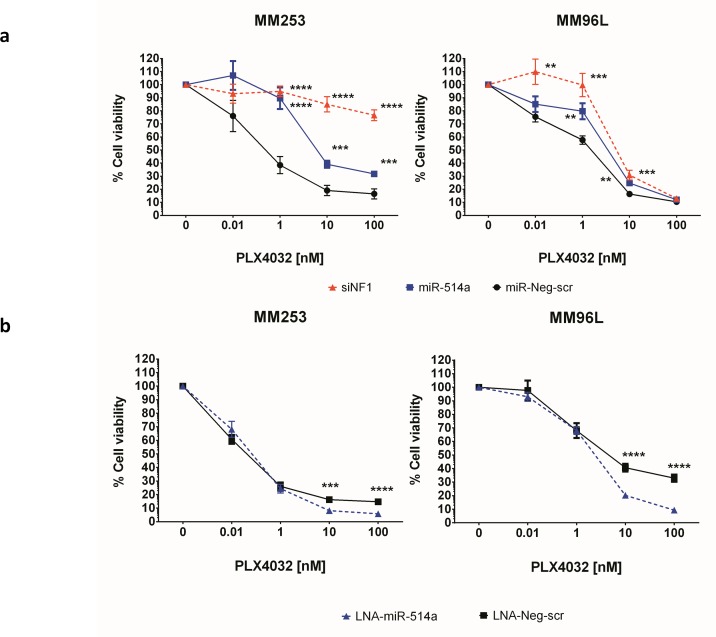
**a.** siNF1, miR-514a-3p (miR-514a), and a negative control (miR-Neg-scr) were transfected into melanoma cell lines MM253 and MM96L with an endogenous *BRAFV600E* mutation. Following 24hr incubation, PLX4032 (BRAFi) was added to all wells (except miR-only or PLX4032 = 0 nM)) with concentrations as indicated. A proliferation assay was performed at day 5 post BRAFi addition. Line graphs show the effect of siNF1 and 514a and miR-Neg-scr respectively, as a % cell viablity in relation to titrated PLX4032 concentrations. **b.** LNA-miR-514a and LNA-Neg-scr were transfected and drug added as in Panel a. Lines graphs show that the cells are more sensitive to BRAFi when miR-514a is inhibited. Error bars show standard error of the mean from 3 biological triplicate experiments.**p < 0.01, ***p < 0.005, ****p < 0.001.

With loss of NF1 [18-21] and more recently an increase of CDK2 (in association with MITF) [22] in melanomas being involved in the resistance to BRAF-inhibitor (BRAFi) therapy, we next sought to investigate whether miR-514a might be involved in this process. Furthermore, cell proliferation rates have also been shown to be involved in BRAFi sensitivity [23]. Firstly, to confirm that NF1 downregulation is involved in inducing BRAFi resistance in MM253 and MM96L (both are BRAFV600E) cell lines, we silenced NF1 expression via specific siRNAs (4x siNF1). We observed > 50% NF1 mRNA knockdown even after 6 days post transfection of siRNAs (data not shown). With NF1 knockdown confirmed, we repeated the experiment with the siRNAs and compared growth rates to cells treated with either miR514a or a scrambled miRNA control (miR-Neg-scr), in the presence of various concentrations of PLX4032 (See Materials and Methods). The siNF1 treatment resulted in significantly increased cell viability compared to miR-Neg-scr (see Supp Table 6 for *P* values) (Figure 5a) with the effect being most apparent in MM253. These data confirm the published association of NF1 loss being involved in reduced BRAFi sensitivity [18-21]. In the presence of miR-514a, cell viability was significantly increased in both MM253 (at 1-100 nM) and MM96L (at 1-10 nM) (see Supp Table 6 for *P* values) when compared to miR-Neg-scr. There was no significant difference in MM96L when siNF1 was compared with miR-514a, whereas in MM253, particularly at higher drug dosage (10-100nM), the differences between siNF1 and miR-514a, were highly significant (*p* < 0.00001) (Figure 5a), indicating that the mode of BRAFi resistance was different, possibly due to other targeted genes of miR-514a. Next we sought to measure the effect of reducing miR-514a levels via a LNA on BRAFi sensitivity. In the presence of PLX4032, using the same LNA specific to miR-514a (LNA-miR-514a), we showed that inhibition of miR-514a resulted in significantly greater BRAFi sensitivity in MM253 and MM96L (Figure 5b) at the 10 (*p* = 0.0002 and *p* = 4.89e-006 respectively) and 100 nM (*p* = 2.21e-005 and *p =* 1.79e-007 respectively) dosage as compared to a negative control (LNA-Neg-scr) (Supp Table 6).

## DISCUSSION

MicroRNAs have been proven to be powerful regulators of protein coding gene expression in almost all facets of cell biology. In cancer, their dysregulation can be far reaching, spanning a multitude of different signaling pathways due to the number of genes being directly and indirectly regulated. In melanoma, the comprehensive analysis of miRNAs has been limited, with most studies performed using only a few hundred miRNAs [2]. Newly characterized miRNAs have seen the expansion of miRBASE (curated miRNA database), largely due to the advent of deep sequencing technologies. As most of the newly identified miRNAs have no known association with melanoma, here we sought to survey a large panel of melanoma cell lines in relation to other solid malignancies to strengthen prior studies into melanocyte-specific miRNAs and to identify those that were previously unknown to be involved in melanomagenesis. Identification of a panel in a comprehensive manner such as this has the ability to open new avenues for research in this burgeoning field, which may provide a greater understanding of this complex disease.

In our analysis we identified 233 miRNAs that were statistically significantly differentially expressed (*P* < 0.05, ≥2 fold) when melanoma (*n* = 55) was compared with other solid cancers (*n* = 34). Several of the identified miRNAs have a known association with melanoma, however a number have no known association. The main premise of the study was to identify a panel of melanocyte-specific miRNAs (or more predominantly melanoma-expressed) and these are more readily observed in the up-regulated group of miRNAs. However, it is worth noting that many of the down-regulated miRNAs have been previously associated with cancers including melanoma. Our dataset confirms and strengthens the associated loss of the miR-200 family (including miR-141) [24, 25] along with the frequently silenced miRNA, miR-205 (Supp Table 2) [26-30].

### Lineage-specific miRNA expression

It has been well documented that miR-211 is a lineage-specific miRNA which is highly expressed in the melanocytic lineage and has been associated with melanoma cell invasiveness [12, 13, 31] via its direct regulation of POU3F2 (BRN2), IGF2R, TGFBR2, NFAT5, and NUAK1. Interestingly, whilst we observe miR-211 to be highly expressed in the melanocytic lineage, as compared to other cancers (highest fold change observed), we have previously reported that miR-211 is often down-regulated in melanoma relative to melanocytes [12]. miR-211 expression is largely driven by its co-regulation with TRPM1 (Melastatin; miR-211 is located in intron 6) via MITF [12, 13, 31], and we have subsequently strengthened this association using an inducible MITF system [14] (Figure 1).

The miRNA with the next highest fold change in expression was miR-514a, which is a member of a cluster of miRNAs on chromosome X (miR-506-514). Most members of this cluster were found to be expressed in melanoma (compared to ‘other’ cancers) along with miR-514a (see Supp Table 1 and 2) however their observed fold changes ranged from 204 (miR-514a) through to 2.5 (miR-513a-5p) and, as such, it could be concluded that they are independently regulated. We assessed whether MITF may play a role in their regulation as was observed with miR-211. Our data suggest that MITF does not directly control the expression of this cluster as we observed only a mild up-regulation in a cell line with endogenous expression and no induced expression in a cell line that had no detectable expression. Therefore, it still remains to be elucidated precisely how this cluster is regulated.

The miR-506-514 cluster has been shown to be involved in melanocyte transformation along with the promotion of melanoma growth [9]. Prior to this study, members of this cluster had also been reported to be over-expressed in melanoma [32], however, we provide the first documented evidence of this cluster being lineage-specific. Streicher *et al*. [9] elegantly showed that the whole cluster was required to initiate cancer formation and its over-expression in melanoma was independent of mutations in BRAF and NRAS (oncogenes highly mutated in melanoma). Whilst we and others have observed the miR-506-514 cluster to be up-regulated in melanoma, individual members of the cluster (miR-506 and miR-507) have been shown to be lost during metastatic colonization despite being up-regulated in early melanoma progression [32].

Collectively, the miR-506-514 cluster is involved in malignant transformation; however we have shown that individual members of the cluster are expressed at different levels. In steps to elucidate the network of genes involved, here we selected miR-514a as it showed the highest fold change difference between melanomas compared with other cancers (204 fold), which was suggestive of its importance within the cluster. In addition, no confirmed gene targets of miR-514a have been reported in melanoma to date. To address this, we performed an unbiased approach (biotin-labeled miRNA duplex ‘pull-down’ of mRNA transcripts) to identify precise target genes, rather than relying on target prediction algorithms which have high false positive rates upon experimental validation [10]. This focused approach yielded a large number of transcripts that were common to two transfected cell lines. Melanoma associated genes were indentified (via key words: melanoma, BRAF, and MITF) which produced a refined gene list (*n* = 16) common to all. One stand-out gene was the tumour-suppressor NF1.

Loss of NF1 function, a well-known tumour-suppressor gene in melanoma, has been implicated on multiple occasions in resistance to PLX4032 targeted therapies [18-21] in BRAF-mutant melanoma. This loss can be attributed to a high frequency of inactivating mutations [33, 34], which occur in ~10% of all melanomas, along with proteasomal degradation, which has been observed in glioma [35, 36]. However, we have now confirmed that miR-514a has the ability to directly bind to *NF1* transcripts, which can lead to altered NF1 protein expression.

Furthermore, as miR-514a is expressed in ~70% of melanoma samples tested, we believe that miR-514a binding could be an important regulator of NF1, and as such warrants further investigation. Moreover, we have also demonstrated that over-expression and inhibition of miR-514a, not only leads to altered NF1 protein expression, but also contributes to altered BRAFi sensitivity *in vitro.* Studies of sequential biopsies taken during response and later at recurrence may reveal that miR-514a does indeed play a key role in the resistance mechanisms observed in melanoma patients undergoing targeted therapy with the common BRAF inhibitor (PLX4032).

It is clear that direct binding of miR-514a to *NF1* is not the sole mechanism this miRNA plays in BRAFi resistance given the number of ‘pull-down’ target genes that are also predicted to be involved (Supp Figure 3) in the process and those as yet to be associated. One of these ‘pull-down’ genes, *CDK2*, was subsequently shown to be induced when miR-514a was inhibited. CDK2 is a cell cycle gene involved in G1/S transition and cell cycle progression. Taken together, these data highlight the important roles that miRNAs play in the control of biological processes. As a single miRNA can control a diverse range of different pathways simultaneously, by inference, a network of miRNAs would therefore have the potential to regulate a multitude of different genes. It could be postulated that targeted-therapy of a few key melanoma-specific miRNAs may allow better management of melanoma progression. In combination with currently used targeted therapies of the MAPK, PI3K, and mTOR pathways, pharmacological intervention of miRNAs may allow for more durable outcomes in late stage melanoma patients.

## MATERIALS AND METHODS

### Cell Culture, Total RNA extraction, microRNA microarray profiling and quantitative RT-PCR validation

Cell lines used along with culturing, STR profiling, RNA extraction, microarray profiling and qRT-PCR are described in the supplementary methods.

### MITF inducible melanoma cell lines and lineage-specific miRNA Taqman Assays

MITF inducible cell lines were cultured, induced with tetracycline, and then harvested for RNA as previously described [14]. Please refer to the supplementary methods for details on quantitative Real-Time PCR.

### Biotin pull-downs and microarray hybridizations and data analysis

Please refer to the supplementary methods for a detailed description of the ‘pull-down’ methodology.

### Site-directed mutagenesis and dual-luciferase reporter assays

Please refer to the supplementary methods for detailed description of constructs used and transfection conditions.

### Transient transfections and cell viability assays

Please refer to supplementary methods for details on miRNA mimics, LNAs, transfection conditions, and BRAFi dosages.

### Western blot and mRNA analysis

Please refer to the supplementary methods for details on protein extraction, western blotting, antibodies, and mRNA primer assays.

## SUPPLEMENTARY MATERIAL AND METHODS TABLES AND FIGURES














