# Supplementary material for: miR-514a regulates the tumour suppressor NF1 and modulates BRAFi sensitivity in melanoma
Source: Oncotarget. 2015 Apr 23;6(19):17753–63. doi: 10.18632/oncotarget.3924 (PMC4627343; doi:10.18632/oncotarget.3924)
Supplement: Supplementary file 1 [file oncotarget-06-17753-s001.pdf]

miR-514a regulates the tumour suppressor NF1 and modulates BRAFi sensitivity in melanoma

Supplementary Material and Methods

Cell Culture and Total RNA extraction

Ethical approval was granted by the QIMR Berghofer's Human Research Ethics Committee (HREC) approval number P1237.

All melanoma (cutaneous and uveal) cell lines (Supp Table 3) were established and have been previously described (Dutton-Regester *et al.*, 2012; Pavey *et al.*, 2004). All other solid cancer cell lines (Supp Table 3) were kind donations from investigators at QIMR Berghofer. Most of the solid cancer cell lines are available from the cell line repositories ATCC or CellBank Australia.

All cell lines were cultured in RPMI (#31800-089, Life Technologies, Foster City, CA, USA) supplemented with 10% FBS (Life Technologies, Foster City, CA, USA), HEPES, 100 U/ ml penicillin and 100 µg/ml streptomycin (Life Technologies, Foster City, CA, USA) and incubated at 37°C (5% CO_2). All cell lines were periodically authenticated via short tandem repeat profiling according to the manufacturer's instructions (AmpFISTR Profiler Plus ID kit; Life Technologies, Foster City, CA, USA). Primary human melanoblasts (QF1160MB) and melanocytes (MELA) were established from human neonatal foreskin and cultured as described (Cook *et al.*, 2003; Leonard *et al.*, 2003). Cells were harvested from the plate and column-purified using the miRNeasy Kit (QIAGEN, Hilden, Germany) according to the manufacturer's instructions.

Serum/plasma collection and Total RNA extraction

Serum and plasma were processed using standard methodologies and Total RNA was extracted using the Plasma/Serum Circulating RNA Purification Kit (#30000; Norgen Biotek, Ontario, Canada) according to manufacturer's instructions.

microRNA microarray profiling and data analysis

5 µg of Total RNA was shipped to LC Sciences (Houston, USA) for miRNA profiling using a custom array platform (µParaflo® technology) containing 1898 miRNAs (miRBase V18)(Griffiths-Jones *et al.*, 2006). All QC, labeling (Cy-3), hybridization, scanning, signal background subtraction and global normalization (LOWESS) were performed by LC Sciences as per technical note: (www.lcsciences.com/documents/application-notes/Tech-Bull-MicroRNA-Microarray-Data-Analysis.pdf).

Advanced data analyses were performed in Genespring GX12.5 (Agilent Technologies, Santa Clara, USA) using the LOWESS normalized signal intensity values. All values <30 were considered 'background expression' (*personal communication* with LC Sciences) and changed to 0.01 prior to $\log_2$ transformation. So as to identify 'melanocyte-specific' miRNAs that were potentially more relevant to melanoma, samples were classified as either 'melanoma' or 'other cancers' (melanocytes, melanoblasts, nevocyte, and serum derived samples were excluded from these categories). To identify differentially expressed miRNAs, a Mann-Whitney U-test (unpaired) was applied to a 'volcano-plot' analysis with thresholds set at $p<0.05$ and ≥2 fold. The gene list derived from these analyses was then applied to all samples in an unsupervised manner using hierarchical clustering (Euclidean similarity with average linkage).

miScript quantitative RT-PCR validation

Briefly, all samples included on the microarray along with an extended cohort of melanoma cell lines (as described in (Boyle *et al.*, 2011)) were reverse transcribed using the miScript II RT Kit (QIAGEN, Hilden, Germany) according to the manufacturer's instructions. Real-time PCR was subsequently performed with a miScript SYBR Green PCR Kit (QIAGEN, Hilden, Germany); with the miRNA primer assays (QIAGEN, Hilden, Germany) hsa-miR-211-5p (#MS00003808), hsa-miR-514-3p (#MS00031948), and RNU-6 (#MS00033740), using the 7900HT Fast Real Time PCR System (Life Technologies, Foster City, CA, USA). Data were analyzed in Microsoft Excel using the ΔCT method compared to RNU6 which was assessed in every sample.

MITF inducible melanoma cell lines and lineage-specific miRNA Taqman Assays

RNA from the MITF inducible cells was used as templates for Taqman miRNA-specific cDNA synthesis as per manufacturer's instructions (Life Technologies, Foster City, USA). Taqman miRNA assays were performed using miRNA-specific primers for miR-211-5p (000514), miR-514a-3p (001147), miR-204-5p (000508), miR-506-5p (001050), miR-508-3p (001052), miR-509-3p (002236), and miR-509-5p (002235) (along with RNU6 (001973) as an endogenous control) using the 7900HT Fast Real Time PCR System (Applied Biosystems, Foster City, USA) as per manufacturer's instructions. Data were analyzed in Microsoft Excel using the ΔCT method compared to RNU6 which was assessed in every sample. The ΔΔCT was then applied with the induced cell line (MITF and LacZ inducible (control)) and the Negative non-induced cell lines (MITF and LacZ non-induced (control)). An 'array expression-like value' was produced by using $\Delta\Delta CT^2$.

Biotin pull-downs and microarray hybridizations and data analysis

Synthetic biotinylated microRNA-duplexes were designed for miR-514a (Sequence 1: /5Phos/rUrArCrUrCrUrGrGrArGrArGrUrGrArCrArArUrCrArCrG Sequence 2: /5Phos/rArUrUrGrArCrArCrUrUrCrUrGrUrGrArGrUrArGrA/3Bio/) along with a scrambled control (Sequence 1: /5Phos/rUrArUrCrCrCrUrUrUrGrCrCrUrGrCrUrUrUrCrC/3Bio/ Sequence 2: /5Phos/rUrArArGrCrUrArGrArCrCrGrGrArGrGrArGrGrGrC) according to specifications detailed in the methodology devised by Cloonan and colleagues (Martin *et al.*, 2014; Wani and Cloonan, 2014) and purchased from Integrated DNA Technologies (Coralville, USA). A step-by-step methodology (Wani and Cloonan, 2014) was followed with the following exceptions optimized to specific melanoma cell lines: Lyophilized miRNA duplexes were resuspended to a final concentration of 50 μM. 500,000 cells in 2.5mls of media (RPMI/10% FBS/no PenStrep) were seeded into 9-wells of 6-well plates (i.e. 1.5 plates). 1.8 μL of each 50 μM duplex (miR-514a and miR-Scr-control (NEG-scr)) was added to 500μL of Opti-MEM® I (Life Technologies, Foster City, USA) and 4 μL of Lipofectamine® 2000 (Life Technologies, Foster City, USA) per well and allowed to incubate for 20mins. 500 μL of transfection mix was then added to each well. Precise modifications of the Cloonan protocol (Wani and Cloonan, 2014) are detailed in the Supplementary Methods below.

Biotinylated cRNA was prepared with the Illumina TotalPrep RNA Amplification Kit (Ambion, Austin, TX, USA). Labelled cRNA was hybridized to HumanHT-12 v4 BeadChip Arrays (Illumina Inc, San Diego, CA, USA), and then washed and scanned according to standard Illumina protocols. Data were extracted in GenomeStudio (Illumina) using default analysis settings and no normalization method. Resulting data were imported

into GeneSpring GX v12.5 (Agilent Technologies, Santa Clara, CA, USA). Expression values were normalized using quantile normalization with default settings.

The gene-lists were filtered firstly by the following criterion: present in at least 2 cell-lines with a fold change of >1.3 fold up-regulated as compared to Neg-Scr. Next, using PubMatrix (http://pubmatrix.grc.nia.nih.gov/) the following keywords were used to find associations: 'Melanoma', 'BRAF', 'MITF', and 'proliferation'.

Biotin pull-downs and microarray step-by-step methods

Biotin pull-downs and microarray hybridizations were modified to work with existing transfection conditions for melanoma cell lines. The protocol follows a step-by-step guide which was originally published by the Cloonan lab (Martin *et al.*, 2014; Wani and Cloonan, 2014).

MATERIALS & REAGENTS

- Cell line of choice
- Fetal Bovine Serum
- Phosphate Buffer Saline
- Opti-MEM ® Reduced Serum Media
- Transfection Reagent
- Biotin tagged miRNA duplexes (Integrated DNA Technologies)
- Dynabeads ® MyOne Streptavidin C1 (Invitrogen cat. no. 650-01)
- Buffer Kit (Ambion cat.no. AM9010)
- Sigma-IGEPAL ® CA-630 (Sigma Aldrich cat. no. I8896)
- DL-Dithiothreitol
- Yeast tRNA (Invitrogen cat.no.15401-011)
- Bovine Serum Albumin (BSA)

• SUPERase•In™ (Ambion cat. no.AM2694)

• Complete Mini Protease Inhibitor EDTA free (Roche cat.no. 11836170001)

• RNase/DNase free water

• RNeasy kit

• Illumina ® TotalPrep RNA Amplification kit (Ambion cat. no. AMIL1791)

• Illumina HumanHT-12 v4 Expression BeadChip.

• Dry Ice

EQUIPMENT

• Cell scrapers

• 6-well tissue culture plates (P6)

• Centrifuge capable of spinning 50mL falcon tubes at 1000g

• 50mL Falcons

• Rotating mixer

• DynaMag™-2 magnet; magnetic separator (Life technologies cat. no. 123-21D)

• Bench top Microcentrifuges; one set at 4°C and one at room temperature, both should be capable of

doing at least 10,000g

• Eppendorf LoBind® tubes; 1.5mL

• Nanodrop 1000 spectrophotometer (Thermo Scientific)

• Illumina Bead Array Reader.

• Filter tips (10ul, 20ul, 200µl and 1000µl)

REAGENT SET UP

• Biotin tagged miRNA duplexes: Resuspend lyophilized miRNA duplexes to a final concentration of

200uM. Make working dilution at 50uM.

▲ CRITICAL Repeat freeze thaw cycles should be avoided.

• Bead Wash Buffer (Once prepared is stable at room temperature for several months):

5mM **Tris-Cl pH 7.5**

0.5mM **EDTA**

1M **NaCl**.

• Solution A (Once prepared is stable at room temperature for several months):

0.1M **NaOH**

0.05M **NaCl** in RNase/DNase free H_2O.

• Solution B (Once prepared is stable at room temperature for several months):

0.1M **NaCl** in RNase/DNase free H_2O.

• Bead Blocking Solution: Prepare RNase/DNase free water containing

1ug/μL **BSA**

1ug/μL **Yeast tRNA**.

▲ CRITICAL This solution must be made fresh on the day of use.

• Cell Lysis Buffer : Prepare a solution with final concentrations of the following in RNase/DNase free

H_2O

10mM KCl,

1.5mM MgCl2,

10mM Tris-Cl pH 7.5,

5mM DTT,

0.5% Sigma-IGEPAL ® CA-630,

60U/ML SUPERase•In and

1x Complete Mini protease inhibitor.

▲ CRITICAL SUPERase In and PIC must be added on the day of use. Then, the solution must be kept on ice at all times.

• Wash Buffer: Prepare a solution with final concentrations of the following in RNase/DNase free

H_2O

10mM KCl,

1.5mM MgCl2,

10mM Tris-Cl pH 7.5,

5mM DTT,

0.5% Sigma-IGEPAL ® CA-630,

60U/ML SUPERase•In,

1x Complete Mini protease inhibitor

▲ CRITICAL This solution must be made fresh on the day of use and must be kept on ice at all

times.

PROCEDURE

Step 1-6: Transfecting the Biotin tagged miRNA duplexes into cells. (Day 1)

● TIMING 24.5 h, 30 mins hands-on

1. For each miRNA to be tested, seed $5x10^5$ cells in 2.5mL media + 10% FCS, no Pen/strep per P6 (at least 8-9 P6 per point tested).

Optional: Set up 1x P6 dish with $5x10^5$ cells as a mock transfection control if desired.

2. Prepare the transfection mix according to the classical Lipofectamine protocol (Mix1 = 1.8ul of miRNA duplex 50uM + 250ul Optimem, Mix2 = 4ul Lipofectamine + 250ul Optimem).

3. Incubate each mix separately for 5min at room temperature.

4. Mix 1 + 2 and Incubate at room temperature for 20min to allow the transfection complexes to form.

5. Add 500ul of the transfection complexes to each of the P6. Swirl the plate gently to ensure that the complexes are distributed uniformly across the plate.

6. Incubate the cells with the transfection complexes at 37ºC and 5% CO_2 for **24 h.**

Step 7-20: Bead washing and blocking. Bead preparation can be started on the day of transfection (day 1) on a rotating mixer at 4º overnight, or can be done on the day of cell harvesting (day 2) for 2 h at room temperature.

● TIMING 17.5 h if started on day 1, 2.5 hs if started on day 2, 30 mins hands-on

7. Resuspend Dynabeads ® MyOne Streptavidin C1 in its bottle by vortexing. Transfer 200µL (**100µL of bead suspension per point**) to a 2mL LoBind tube.

8. Place the tube with the bead suspension on the DynaMag™-2 magnet for 2 minutes.

9. Using a pipette, aspirate and discard supernatant before removing tube from the DynaMag™-2 magnets.

10. Add **200µl of bead wash buffer** to the beads. Pipette several times to ensure the beads are washed sufficiently.

11. Repeat steps 8 -10 twice more for a total of three washes.

12. RNase Freeing Beads: After the third wash resuspend the beads in **200µl Solution A**. Mix well by pipetting several times. Let beads incubate **at room temperature for 2 minutes**.

13. Place the tube containing the bead solution on the DynaMag™-2 magnet for 2 minutes.

14. Using a pipette, aspirate and discard supernatant before removing tube from the DynaMag™-2 magnets.

15. Repeat steps 12-14 once more.

16. Resuspend beads in **200µl Solution B**. Mix well by pipetting several times.

17. Place the tube containing the bead solution on the DynaMag™-2 magnet for 2 minutes.

18. Using a pipette, aspirate and discard supernatant before removing tube from the DynaMag™-2 magnets.

19. Resuspend beads in **400µl Bead blocking solution**. Pipette several times to mix.

20. Place the tube containing the bead **on a rotating mixer at 4ºC overnight** if starting on day 1, or

allow mixing at room temperature for 2h if starting on day 2.

Steps 21- 36 Harvesting and lysing transfected cells (Day 2)

● TIMING 1 h , 30 mins hands-on

21. Prepare cell Lysis Buffer and keep buffer on ice till required.

▲ CRITICAL this buffer must be made fresh on the day of use.

22. Retrieve transfection plates from step 6 of the protocol. Aspirate and discard media.

23. To each P6 well, add 1 mL of PBS and using a cell scraper gently lift off the cells from the plate.

24. Using a pipette transfer the cells to a 50mL falcon tube. Note: Cells from each of the 9xP6 should be pooled at this stage.

25. Centrifuge the falcon tubes containing the harvested cells at 1800rpm for 5 minutes at room temperature.

26. Aspirate and discard the supernatant. Add 1mL of PBS to the cell pellet and then pipette gently to wash and resuspend the cells. Transfer into a 1.5ml Eppendorf tube.

27. Centrifuge the tube at 10 000rpm for 5 minutes at room temperature.

28. Aspirate and discard supernatant.

29. Add 150μl cell lysis buffer to each pellet and gently pipette to resuspend cells.

30. Put cells in lysis buffer at -80°C for 20 mins.

31. Allow the cells to thaw out at room temperature.

▲ CRITICAL this freeze thaw step allows for better lysis of cells.

32. Centrifuge tube at 13,000rpm in a bench top centrifuge set at 4ºC for 5 minutes.

33. Transfer the cleared cell lysate to a clean 1.5mL LoBind tube, leaving behind the soft pellet. The final volume of cleared lysate should be ~140μl-150ul.

34. Transfer 20μL of this cleared lysate to a clean 1.5mL LoBind tube and keep on ice. This lysate will serve as the control lysate RNA which can be extracted with the miRNeasy mini kit (elution in 30μl).

Note: If processing a mock transfected control, carry out steps 22-33 of protocol on the mock transfection plate and keep mock control lysate on ice for later use.

35. Add **NaCl** to the cleared lysate to give a final concentration of **1M**. For example, add 32.5μL of

5M NaCl to 130μL of lysate, giving a final concentration of 1M NaCl in 162.5ul.

36. Place the tube containing cleared lysate and NaCl on ice.

Steps 37-44 Bead Preparation

● TIMING 15 mins, 15 mins hands-on

37. Prepare wash Buffer and keep buffer on ice till required.

▲ CRITICAL This buffer must be made fresh on the day of use.

38. Place the tube with the beads from step 20 on the DynaMag™-2 magnets for 2 minutes.

39. Aspirate and discard supernatant using a pipette.

40. Add **200µl of wash buffer** and Resuspend beads by pipetting several times.

41. Place tube on the DynaMag™-2 magnets for 2 minutes.

42. Aspirate the supernatant using a pipette and discard. Remove the tube from the DynaMag™-2

magnets.

43. Repeat steps 40-42

44. Resuspend beads in **600µl of wash buffer**.

Steps 45-52 Target mRNA capture and post capture bead washing

● TIMING 45 mins, 15 mins hands-on

45. Add the **130µl cell lysate + NaCl** from step 36 to the **100µl prepared beads** from step 45. Place tubes **on a rotating mixer at room temperature and incubate for 45 minutes**.

46. Place the tube containing the cell lysate + Beads on the DynaMag™-2 magnets for 2 minutes.

47. Aspirate supernatant using pipette and discard. Remove the tube from the DynaMag™-2 magnets.

48. Wash beads in **100µl wash buffer**. Mix well by pipetting several times.

49. Place the tube containing Beads + wash buffer on the DynaMag™-2 magnets for 2 minutes.

50. Aspirate supernatant using pipette and discard. Remove the tube from the DynaMag™-2 magnets.

51. Repeat steps 48 -50 <u>three times</u>.

▲ CRITICAL These washes are essential and are important for the removal of non-specifically bound products from the beads.

52. Once the washes are complete, resuspend the beads in **30µL of RNase/DNase free H_2O**, store on ice and proceed immediately to the next step.

▲ CRITICAL The target mRNAs are captured on these beads. Do not discard.

Steps 53-59 Target mRNA and control lysate RNA purification

● TIMING 1 h, 30 mins hands-on

53. Target mRNAs are purified off the Dynabeads® MyOne Streptavidin C1 using a **Qiagen RNeasy kit** according to manufacturer's RNA clean-up protocol. RNA should be eluted twice in 30µl RNase/DNase free water. The final volume of RNA will be 60µl.

54. Alongside the captured target mRNAs also purify the control lysate RNA from step 34 of this protocol. Make up the volume of the control lysate to 100µL by adding 90µL of RNase /DNase free water. Purify the control samples using a Qiagen miRNeasy kit according to manufacturer's RNA clean-up protocol. The control RNA can be eluted in 30-50µL of RNase/DNase free water.

■ PAUSE POINT Samples can now be stored at -80ºC. Alternatively, continue with quantification of RNA.

Step 60 Target mRNA and control RNA Quantification

● TIMING 45mins, 20 mins hands-on

60. The target mRNA and the control lysate RNA can now be quantified on the Nanodrop 1000 spectrophotometer and on an Agilent 2100 Bioanalyser using the Agilent RNA 6000 Pico kit using manufacturer's protocol.

■ PAUSE POINT Samples can now be stored at -80ºC or continue on with RNA amplification.

Step 61- 62 Target mRNA Labeling and Amplification

● TIMING 23 h, 2 h hands-on

61. Amplify and label 160-500ng of captured target mRNA using Illumina® TotalPrep RNA Amplification kit according to manufacturer's instructions. Also amplify and label 160-500 ng of control RNA.

▲ CRITICAL Carry out the 16 hour incubation for the IVT step.

62. Quantify the amplified RNAs on the Nanodrop 1000 spectrophotometer.

Step 63-65 Hybridization of samples onto Illumina® Human HT-12 array and scanning

● TIMING 18 h s, 2 h s hands-on

63. Hybridize 750ng of amplified cRNA onto an Illumina® Human HT-12 array following manufacturer's protocol.

64. Scan the arrays using an Illumina BeadArray Reader.

65. Extract the expression measurements using the GenomeStudio software.

Site-directed mutagenesis and dual-luciferase reporter assays

Putative binding sites were identified using the miRanda target prediction software (version August 2010)(Enright *et al.*, 2003). A partial sequence of the cDNA of NF1 (NF1; NM_000267) from positions 1279–3542, which includes two putative miR-514a-binding sites (site 1 in exon 9, 1342-1360bp; site 2 in exon 23, 3420– 3435 bp), flanked by *AvrII* sites, was cloned into the pGL4.10 vector (Promega, Madison, WI, USA) downstream of the luciferase gene. Site-directed mutagenesis of the putative binding sites was carried out using a QuickChange II XL site-directed mutagenesis kit (Stratagene, CA, USA). Melanoma cell lines with endogenous expression of miR-514a (C-32, MM253, and HT144) were seeded onto 96-well plates at 25 000 cells per well and

co-transfected with 400 ng of pGL4.10 NF1 WT, Ex9_Mut, Ex23_Mut or both vector and 20 ng pGL4.75 expressing Renilla luciferase. Luciferase activity was measured using the Dual-Luciferase Reporter Assay System as per the manufacturer's instructions (Promega, Madison, WI, USA). Firefly luciferase activity was normalized to corresponding Renilla luciferase activity and expressed as a percentage of the control. To confirm that the mutated binding sites were related to miR-514a expression, MM253 and HEK293T was co-transfected with 5 nM of miR-514a-3p mimic or Negative Allstars control (miR-Neg-scr) (QIAGEN, Hilden, Germany) along with 400 ng of pGL4.10 NF1 WT and pGL4.10 NF1 Mut (Ex9 and Ex23) and 20 ng pGL4.75 expressing Renilla luciferase.

Transient transfection of miRNA mimics, miRNA inhibitors, siRNAs and cell viability assays

A highly efficient transfection reagent allows the use of low amounts of mimic thus reducing the possibility of off-target effects. Adding too much miRNA mimic can overload the RNA-induced silencing complex (RISC)(Khan *et al.*, 2009)

miR-514a-3p mimic (#MSY0002883), Negative Allstars control (#1027280; miR-NEG-scr), and siRNAs (FlexiTube GeneSolution GS4763 for NF1) were purchased from QIAGEN (Hilden, Germany). miRNA inhibitors (miRCURY LNA™ microRNA Power Inhibitors # 427206-04 and 199020-04) were purchased from Exiqon (Vedbaek, Denmark). A final concentration of 5 nM of mimic, siRNAs and Negative control and 50 nM of LNA's were reverse-transfected into melanoma cell lines (30,000 cells/6-well and 4,000 cells/well; MM96L and MM253 using Lipofectamine® RNAiMAX (Life Technologies, Foster City, USA) and harvested for RNA, protein at 72 hrs or measured for cell viabilities 6-days post transfection (equal to 5 days post BRAFi addition).

Melanoma cell lines were selected based upon transfection ability along with having detectable (by Western blot) endogenous NF1 protein levels (data not shown). NF1 mRNA expression was determined (relative to RNU6) using a QuantiTect Primer Assay (#QT00065016; QIAGEN) as described previously. Cell viability assays were performed and determined using a modified sulforhodamine B (SRB; Sigma, St Louis, USA) assay (Vichai and Kirtikara, 2006). Briefly, siRNAs, miRNA mimic, Negative control or LNA's were reverse-transfected with melanoma cell lines and seeded into a 96-well plate then incubated at 37ºC with 5% CO_2 for 24hr. A serial 10-fold dilution series (100 nM-0.01 nM) of PLX4032 (Selleckchem, Houston, USA) in DMSO was added across each plate. Plates were fixed on day 6 with methylated spirits prior to performing the SRB assay and read at 564 nm using a plate reader (Molecular Devices, Sunnyvale, USA).

Western blot and mRNA analysis

Cells were lysed in ice-cold lysis buffer containing 20 mM HEPES, pH 7.8, 0.42 M NaCl, 0.5% NP40, 25% glycerol, 0.2 mM EDTA, 1.5 mM MgCl2, 0.1 mM PMSF and protease and phosphatase inhibitors. Samples (60 µg total protein) were resolved on 4–15% Mini-PROTEAN TGX gels (Bio-Rad, Hercules, USA) and transferred to PVDF membranes using a Trans-Blot®Turbo™ (Bio-Rad, Hercules, USA). The following antibodies were used to detect NF1 (#A300-140A; Bethyl Laboratories, Montgomery, USA) and GAPDH (#2118S, Cell Signaling Technology, Danvars, USA) at 1:1000 and 1:5000 dilutions respectively along with a HRP-linked anti-rabbit secondary at 1:2000 (#7074S, Cell Signaling Technology, Danvars, USA). Cell-cycle and apoptosis related proteins were detected with the following antibodies: rabbit anti-E2F1 (#3742S), rabbit anti-CDK2 (#2546S), mouse anti-cyclin D1 (#2926P) and rabbit anti-BCL2 #2876S (Cell Signaling Technology, Danvars, USA), at 1:1000 dilution. Enhanced chemiluminescence (ECL)

detection of antibody binding was quantified using the Fuji LAS-4000 (GE Healthcare Life Sciences, Buckinghamshire, England).

NF1 mRNA expression analysis

RNA was reverse transcribed using the miScript II RT Kit (QIAGEN, Hilden, Germany) using the 'HiFlex' Buffer thus enabling both mRNA and miRNA to be analyzed in the same sample. The primer assays were used as described NF1 (#QT00065016) along with (RNU-6). Real-time PCR was performed and analyzed as previously stated.

References

Boyle GM, Woods SL, Bonazzi VF*, et al.* (2011) Melanoma cell invasiveness is regulated by miR-211 suppression of the BRN2 transcription factor. *Pigment Cell Melanoma Res* 24:525-37.

Cook AL, Donatien PD, Smith AG*, et al.* (2003) Human melanoblasts in culture: expression of BRN2 and synergistic regulation by fibroblast growth factor-2, stem cell factor, and endothelin-3. *J Invest Dermatol* 121:1150-9.

Dutton-Regester K, Aoude LG, Nancarrow DJ*, et al.* (2012) Identification of TFG (TRK-fused gene) as a putative metastatic melanoma tumor suppressor gene. *Genes Chromosomes Cancer* 51:452-61.

Enright AJ, John B, Gaul U*, et al.* (2003) MicroRNA targets in Drosophila. *Genome Biol* 5:R1.

Griffiths-Jones S, Grocock RJ, van Dongen S*, et al.* (2006) miRBase: microRNA sequences, targets and gene nomenclature. *Nucleic Acids Res* 34:D140-4.

Khan AA, Betel D, Miller ML*, et al.* (2009) Transfection of small RNAs globally perturbs gene regulation by endogenous microRNAs. *Nat Biotechnol* 27:549-55.

Leonard JH, Marks LH, Chen W, *et al.* (2003) Screening of human primary melanocytes of defined melanocortin-1 receptor genotype: pigmentation marker, ultrastructural and UV-survival studies. *Pigment Cell Res* 16:198-207.

Martin HC, Wani S, Steptoe AL, *et al.* (2014) Imperfect centered miRNA binding sites are common and can mediate repression of target mRNAs. *Genome Biol* 15:R51.

Pavey S, Johansson P, Packer L, *et al.* (2004) Microarray expression profiling in melanoma reveals a BRAF mutation signature. *Oncogene* 23:4060-7.

Vichai V, Kirtikara K (2006) Sulforhodamine B colorimetric assay for cytotoxicity screening. *Nature protocols* 1:1112-6.

Wani S, Cloonan N (2014) Profiling direct mRNA-microRNA interactions using synthetic biotinylated microRNA-duplexes. *bioRxiv*.

SUPPLEMENTARY TABLE LEGENDS

Supplementary Table 1

All miRNA genes (n=233) listed have a P value ≤0.05 with a fold change (FC) ≥2 when 55 melanoma cell lines were compared with 34 other solid malignancies. P values were generated using a Mann-Whitney U test (unpaired) with multiple testing corrections

(Benjamini-Hochberg). Normalised expression data of 233 miRNAs genes in all samples present on the microarray. Samples are grouped based upon tissue type. Normalised expression data is conditionally formatted (in Microsoft Excel) to assist in data visualisation.

Supplementary Table 2

All miRNAs genes that were ≥10 fold up and downregulated from those listed in Supplementary Table 1 are summarized here along with their relevance to melanoma.

Supplementary Table 3

Summary of all cell line names and associated tissue type that were used in the discovery (microarray) and validation cohorts (qRT-PCR).

Supplementary Table 4

List of all genes that we upregulated in 2/2 melanoma cell lines following a biotin-labelled miR-514a duplex pulldown of mRNA transcripts.

Supplementary Table 5

List of all possible binding sites of miR-514 in the NF1 transcript (5'UTR, coding, and 3'UTR) using the miRanda prediction algorithm. Binding threshold is set to 100 (default=140).

Supplementary Table 6

Table of *P* values associated with BRAFi sensitivity for the melanoma cell lines MM96L and MM253 (Figure 5A-B). T-tests were performed with comparisons were made between siNF1 vs miR-514a; siNF1 vs siNF1+miR-514a; siNF1 vs miR-Neg-scr; miR-

514a vs miR-Neg-scr; LNA-miR-514a vs LNA-Neg-scr. All tests were corrected for multiple comparisons using the Sidak-Bonferroni method and those that still remained significant are noted (*).

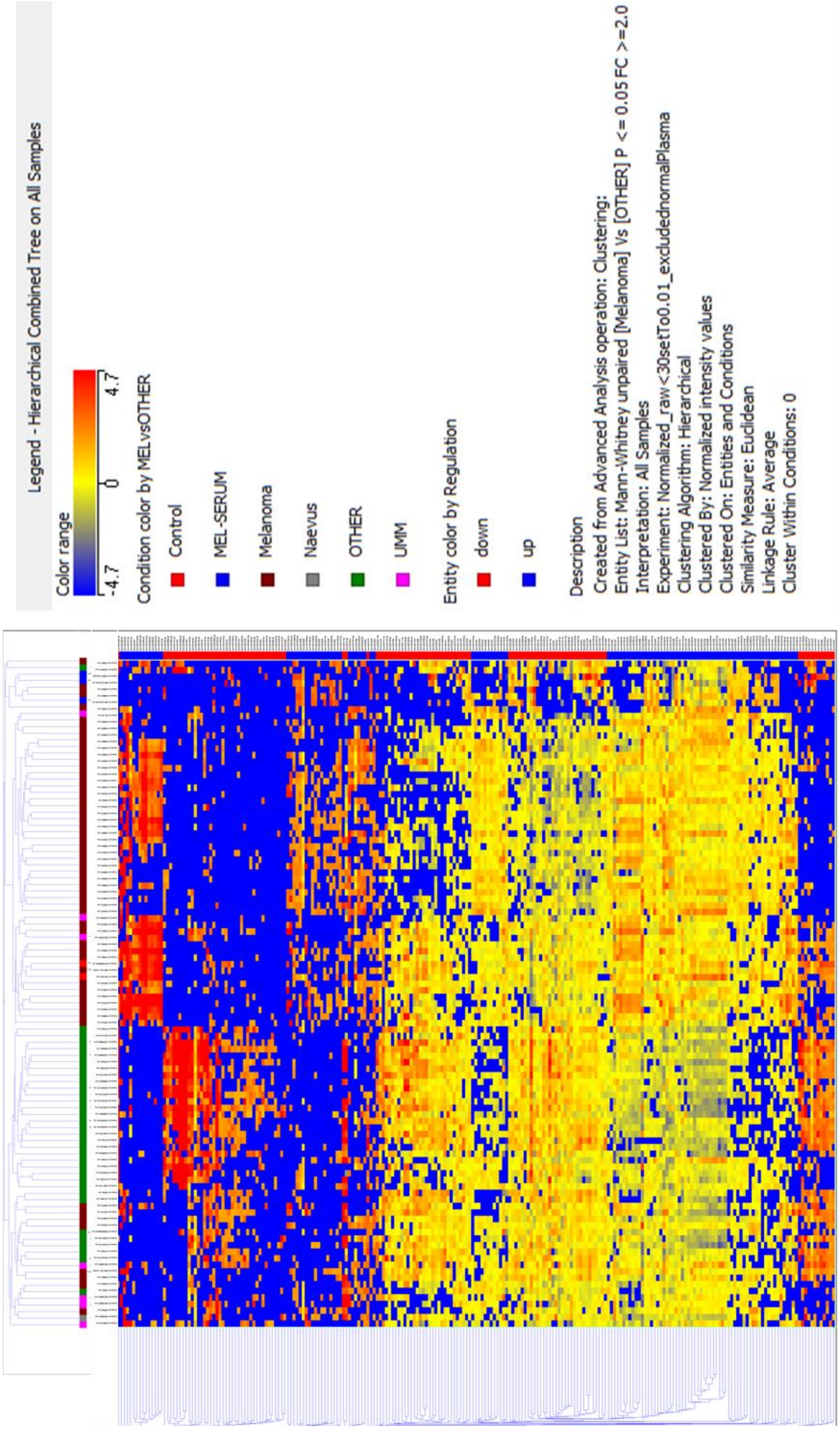

SUPPLEMENTARY FIGURE 1

Unsupervised hierarchical cluster tree (Euclidean similarity with average linkage) of all miRNA genes and samples (Supplementary Table 1). Distinct separation can be

observed for most melanoma (cutaneous) samples (brown) compared with other solid malignancies (green). Controls (melanocytes and melanoblsts) (red), uveal melanoma (UMM) (pink), melanoma patient-derived serum (blue) and nevocyte (grey) are also present in the tree.

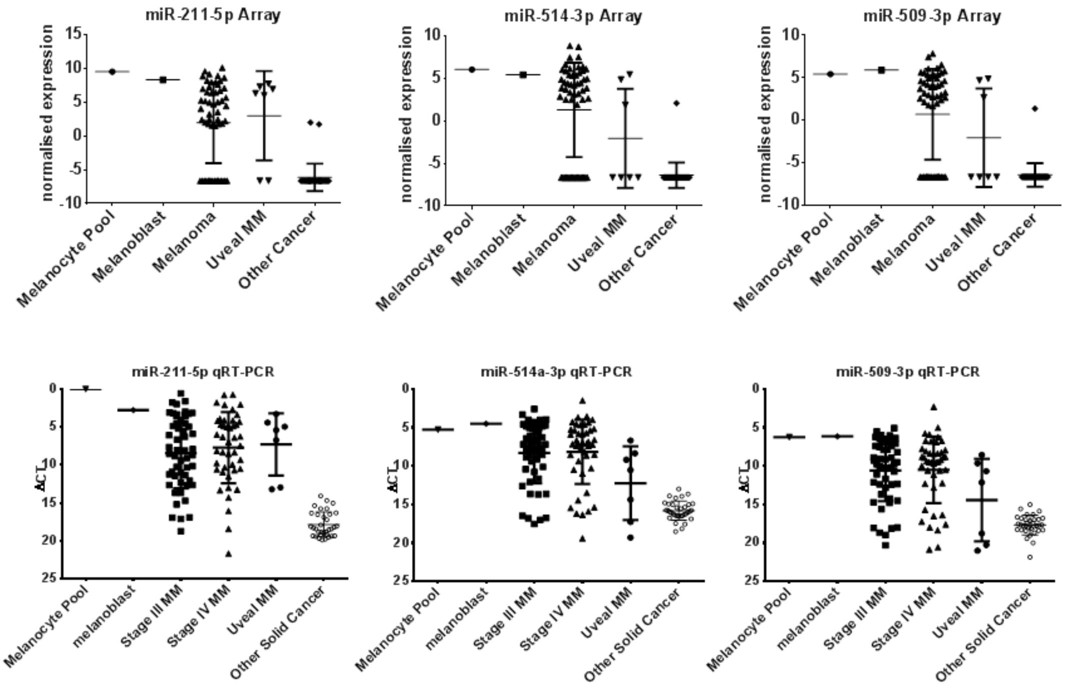

SUPPLEMENTARY FIGURE 2

The top 3 upregulated miRNAs were validated using qRT-PCR with all samples present on the microarray along with an extended cohort of melanoma cell lines (Supplementary Table 3). For comparative purposes the array data was also plotted to highlight the high correlation between the array and qRT-PCR.

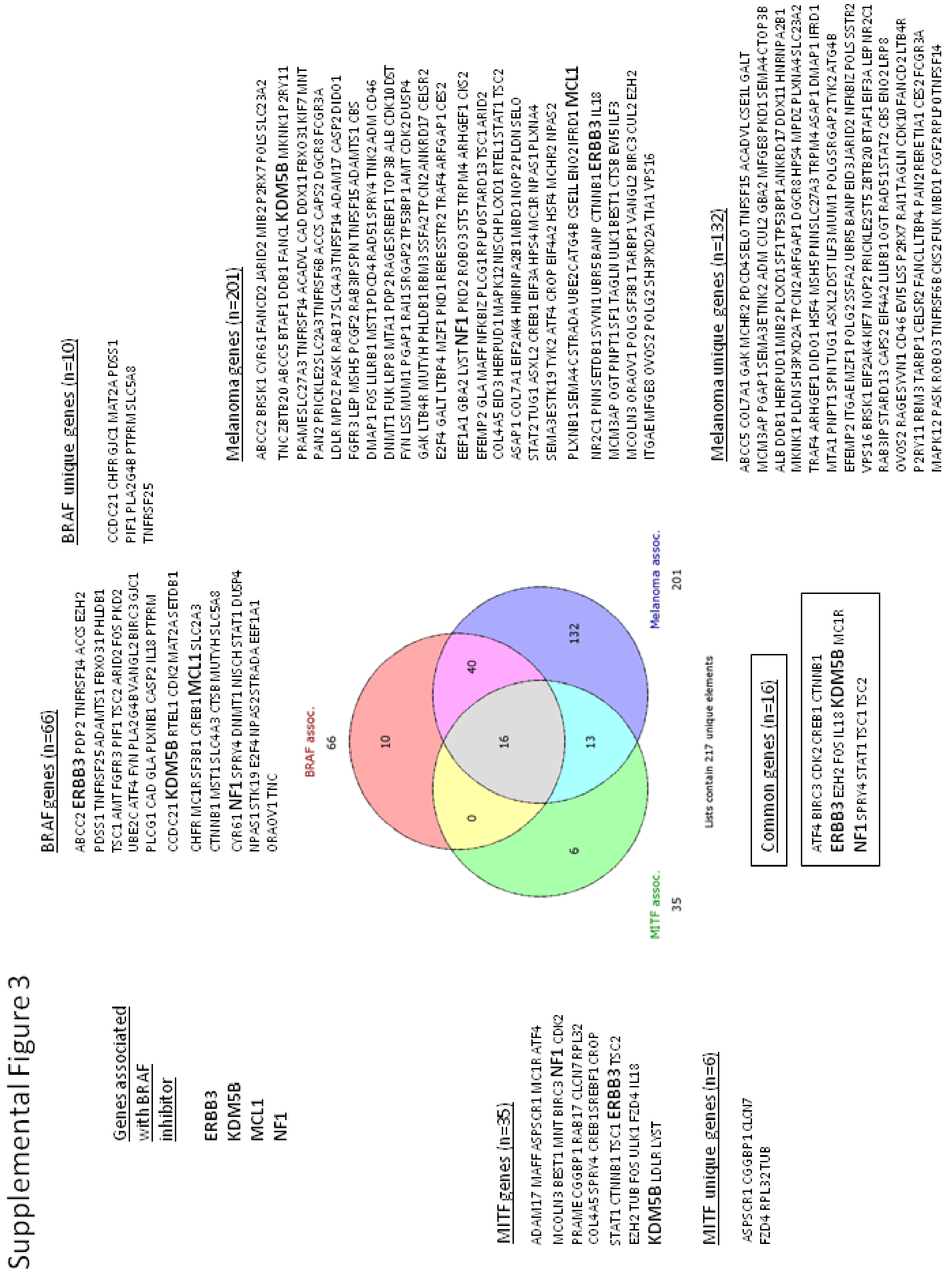

SUPPLEMENTARY FIGURE 3

The Venn diagram graphically represents the filtered gene lists derived from keyword searches ('Melanoma', 'BRAF', 'MITF') compared to the common 2/2 gene list (Supplementary Table 4).
